# Left Ventricular Reverse Remodeling after Surgical Aortic Valve Replacement for Aortic Regurgitation—An Explorative Study

**DOI:** 10.3390/diseases12080191

**Published:** 2024-08-21

**Authors:** Zsuzsanna Arnold, Alexander Elnekheli, Daniela Geisler, Thomas Aschacher, Verena Lenz, Bernhard Winkler, Reinhard Moidl, Martin Grabenwöger

**Affiliations:** 1 Department of Cardiovascular Surgery, Clinic Floridsdorf, 1210 Vienna, Austria; daniela.geisler@gesundheitsverbund.at (D.G.); thomas.aschacher@gesundheitsverbund.at (T.A.); verena.lenz@gesundheitsverbund.at (V.L.); bernhard.winkler@gesundheitsverbund.at (B.W.); reinhard.moidl@gesundheitsverbund.at (R.M.); martin.grabenwoeger@gesundheitsverbund.at (M.G.); 2 Institute of Cardiovascular Research, Karl Landsteiner Society, 1210 Vienna, Austria; 3 Medical Faculty, Sigmund Freud University, 1020 Vienna, Austria

**Keywords:** reverse remodeling, aortic regurgitation, biological valve, mechanical valve

## Abstract

Background: The timing of treatment for chronic aortic valve regurgitation (AR), especially in asymptomatic patients, is gaining attention since less invasive strategies have become available. The aim of the present study was to evaluate left ventricular reverse remodeling after aortic valve replacement (AVR) for severe AR. Methods: Patients (n = 25) who underwent surgical AVR for severe AR with left ventricular ejection fraction (LVEF) less than 55% were included in this study. Preprocedural and follow-up clinical and echocardiographic measurements of LVEF and left ventricular (LV) diameters were retrospectively analyzed. Results: Mean LVEF increased significantly following surgical AVR (*p* < 0.0001). LV diameters showed a clear regression (*p* = 0.0088). Younger patients and those receiving a mechanical valve tended to have less improved LVEF on follow-up than patients over 60 years or the ones who were implanted with a biological prosthesis (*p* = 0.0239 and *p* = 0.069, respectively). Gender had no effect on the degree of LVEF improvement (*p* = 0.4908). Conclusions: We demonstrated significant LV reverse remodeling following AVR for AR. However, more data are needed on LV functional and geometrical improvement comparing the different types of valve prostheses to provide an optimal treatment strategy.

## 1. Introduction

With the growing use of transcatheter aortic valve implantation (TAVI) for treating combined aortic valve disease, the timing of valve replacement in patients with chronic aortic regurgitation (AR) has garnered renewed attention [1,2]. In particular, asymptomatic patients with early signs of left ventricular (LV) distress are the focus of interest [3]. Adverse LV remodeling resulting from long-lasting volume overload manifests as a drop in performance, dyspnea, and palpitations through to angina pectoris. Left untreated, the structural and functional changes in the heart lead to consecutive heart failure, with increasingly frequent hospitalizations [4].

Current guidelines recommend surgical treatment based on the following criteria: presence of symptoms, severity of AR, LV ejection fraction (LVEF), and LV diameter. In case of surgery for another valve or coronary artery disease, treatment of the regurgitant aortic valve is proposed for patients with moderate [5] or severe [6] AR. Thus, the asymptomatic patient with severe AR but preserved LV dimensions awaits the deterioration of cardiac function and geometry or another cardiac pathology to develop. Whether the criteria provided in the guidelines or their cutoff values for the indication of surgical intervention should be revised has been controversially discussed in recently published studies [7,8,9,10]. Furthermore, the 2021 European guidelines on valvular heart disease list asymptomatic AR as one important area with gaps in evidence, especially in terms of the timing of treatment [6].

Adverse remodeling of the LV has been studied extensively; on the other hand, much less is known about the extent to which geometric and functional deterioration of the LV may be reversed after surgical aortic valve replacement (AVR) [11,12,13]. Moreover, a comparison of different aortic valve prostheses from this point of view is lacking.

We aimed to evaluate clinical outcomes and LV reverse remodeling following AVR for acute or chronic AR.

## 2. Materials and Methods

This retrospective study was approved by the local ethics committee (Ethics Committee Vote Number: EK 22-033-VK).

### 2.1. Study Population

Patients with reduced left ventricular systolic function (LVEF ≤ 55%) who underwent open surgical AVR for severe AR in our department between April 2005 and August 2020 were included in the study. Asymptomatic and symptomatic patients as well as those with a history of previous cardiac surgery or infectious endocarditis were also considered to be eligible for the study. Subjects with multiple valve diseases, coronary artery disease, or any other condition potentially responsible for cardiomyopathy were removed from the analysis. For the purpose of this study, we confined the analysis to procedures involving prosthetic replacement of the aortic valve; accordingly, aortic valve-sparing procedures were not investigated in this research. Participants with inadequate echocardiographic assessments were excluded. The type of implanted valve prosthesis was recommended according to the current guidelines and the decision was made by the patient upon informed consent.

New York Heart Association (NYHA) functional class for dyspnea was applied to assess the severity of symptoms [14]. Operative mortality risk was defined using the European System for Cardiac Operative Risk Evaluation (EuroSCORE) II score [15]. In-hospital data were gathered retrospectively. Cross-sectional follow-up was conducted actively for a minimum of one year after the index procedure by direct contact with patients or by phone calls using a structured questionnaire and supported by a passive review of electronic medical records. An autopsy report or documentation of the cause of death was available in 100% of the mortalities.

### 2.2. Echocardiographic Assessment

Echocardiography was performed preprocedural in all cases. Follow-up echocardiographs were obtained either in our outpatient clinic or by the patient’s cardiologist and requested via phone call or e-mail. Data on aortic valve morphology and function, LV dimensions and function, as well as mean and peak pressure gradients (MPG and PPG, respectively), maximal transvalvular velocity, and effective orifice area, if available, were analyzed.

### 2.3. Study Endpoints

The primary endpoints included LV structural and functional parameters obtained by echocardiography at least six months after AVR. As secondary endpoints, early and mid-term mortality and morbidity, including major adverse cardiac and cerebrovascular events (MACCE) and structural valve deterioration (SVD), were declared. MACCE was defined as cardiac mortality, myocardial infarction, coronary revascularization, stroke, and hospitalization because of heart failure. Functional improvement was assessed based on the NYHA class. Reverse remodeling of the LV was further evaluated according to the type and size of the surgical valve prosthesis, prosthesis–patient mismatch (PPM), age, and gender of the patient.

Morbidity and mortality, including valve-related events and parameters such as structural and non-structural valve deterioration, PPM, prosthetic valve endocarditis, and early and valve-related mortality were defined and reported according to contemporary guidelines and related consensus statements [16,17,18].

### 2.4. Statistical Analysis

Categorical variables are reported as frequency (percentage), whereas continuous variables are reported as mean ± standard deviation (SD). Comparisons of preprocedural and follow-up echocardiographic data were calculated using paired Student´s *t*-test or Wilcoxon matched-pairs signed rank test in case of non-normal distributions. We performed two-way analyses of variances (two-way ANOVA or General Linear Model) when comparing different groups and Tukey’s multiple comparisons test was used to adjust the *p*-value. A *p*-value < 0.05 was considered significant and all analyses were computed as two-tailed. IBM SPSS Statistics for Windows, version 27 (IBM Corp., Armonk, NY, USA), and GraphPad Prism version 9.3.1 (for Windows, GraphPad Software, San Diego, CA, USA, www.graphpad.com) software were used for statistical analysis.

## 3. Results

### 3.1. Procedural Outcome

Between April 2005 and August 2020, a total of 25 patients fitting the inclusion and exclusion criteria received a biological or mechanical surgical AVR for isolated AR at our department.


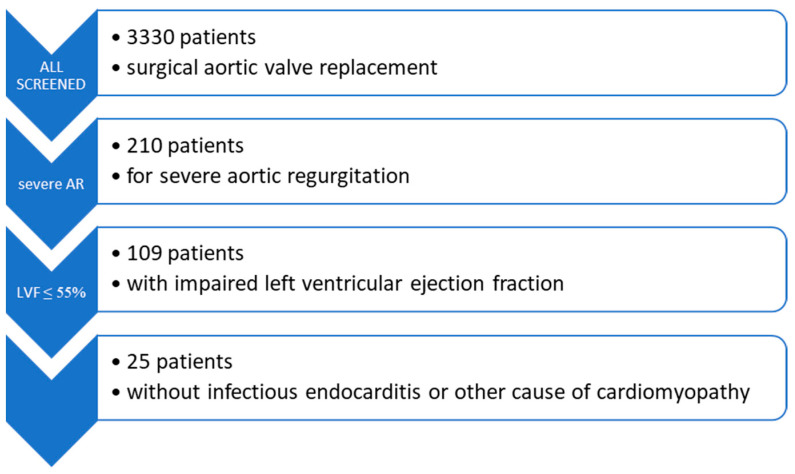


Baseline demographics and procedural characteristics are given in Table 1.

One patient with acute type A dissection of the aorta died on postoperative day 24 due to multiorgan failure after the emergency index operation and pericardial drainage on postoperative day 15. Two permanent pacemakers (8%) needed to be implanted and five patients (including the one with a fatal outcome) were ventilated longer than 72 h (20%). De novo atrial fibrillation was documented in five cases (20%) and reoperation due to bleeding or pericardial drainage was needed in three cases (12%). Eleven patients (44%) were transfused with red blood cells. None of the patients suffered a neurological event. Thus, the overall 30-day MACCE rate was 4% (n = 1).

### 3.2. Early Hemodynamics

Discharge echocardiographs demonstrated regular hemodynamics with a mean PPG of 23.2 ± 6.6 mmHg and a mean MPG of 13.6 ± 4.3 mmHg. Implanted valve sizes ranged from 21 mm (n = 2), through 23 mm (n = 3) and 25 mm (n = 9), to 27 mm (n = 11). The implanted models as well as concomitant procedures are listed in Table 2. Only one moderate and no severe PPM was detected.

### 3.3. Mid-Term Clinical Outcome

Follow-up was 100% complete, at a mean of 2.9 ± 3.4 years, summing up to 61.5 patient-years. Two further patients died during follow-up due to non-valve-related causes. Causes of late death included one sepsis and one liver failure. One other patient needed rehospitalization due to suspected prosthesis endocarditis. Signs and symptoms resolved upon conservative therapy. No thromboembolic or neurological events were observed throughout the follow-up period. Consequently, the MACCE rate during follow-up was 4.2% (n = 1 out of 24). All patients alive reported a stable NYHA I functional class. One of the implanted biological prostheses showed early signs of moderate SVD with a 10 mmHg increase in MPG.

### 3.4. Left Ventricular Reverse Remodeling

**Figure 1:**
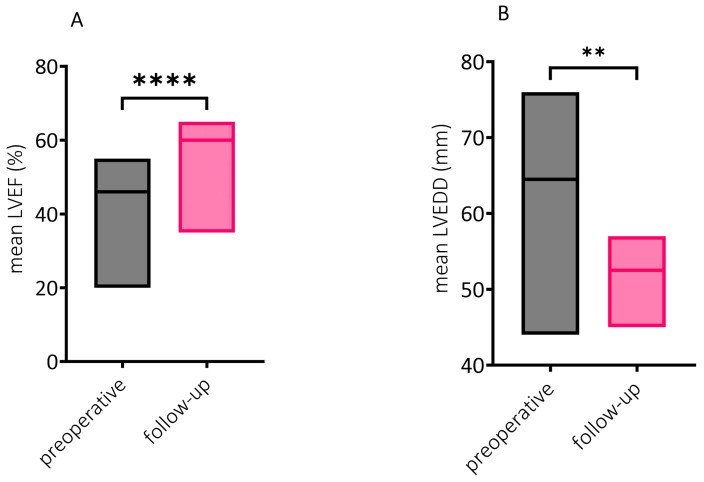
Echocardiographic parameters of left ventricular reverse remodeling. Significantly improved left ventricular ejection fraction (LVEF) upon follow-up (mean LVEF 59.1 ± 5.6% vs. 44.7 ± 8.4%, respectively; **** *p* < 0.0001; n = 21 pairs) in comparison to preoperative LVEF. Due to the skewed distribution of the data, the Wilcoxon matched-pairs signed rank test was used (**A**). Significantly decreased left ventricular end-diastolic diameter (LVEDD) at follow-up compared to preoperative measurements (52.7 ± 3.9 mm vs. 67.5 ± 5.0 mm, respectively; ** *p* = 0.0088; n = 6 pairs). Paired *t*-test was performed (**B**). Results are shown as mean ± standard deviation (SD).

Figure 1A shows the extent of left ventricular ejection fraction (LVEF) improvement at least six months after the index procedure compared to preoperative LVEF (59.1 ± 5.6% vs. 44.7 ± 8.4%, respectively; *p* < 0.0001). Furthermore, as a sign of left ventricular remodeling, left ventricular end-diastolic diameter (LVEDD) also decreased significantly (52.7 ± 3.9 mm at follow-up vs. 67.5 ± 5.0 mm preoperatively; *p* = 0.0088), as seen in Figure 1B.

**Figure 2:**
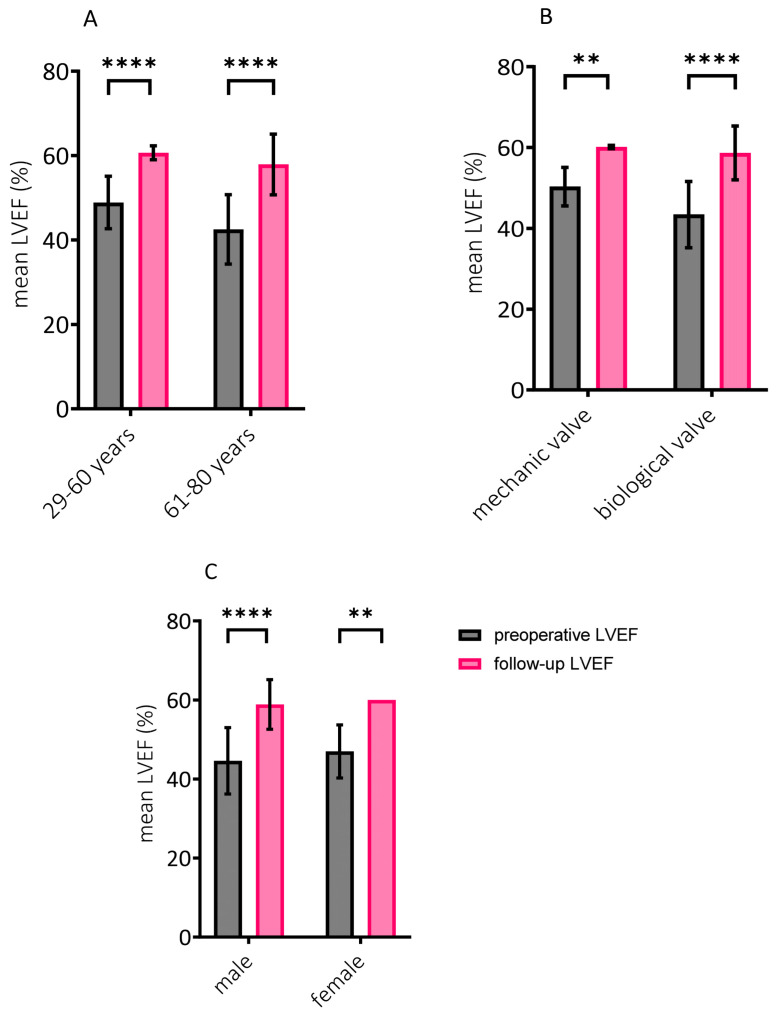
Improvement of left ventricular ejection fraction analyzed according to age, prosthesis type, and gender. Significant improvement in left ventricular ejection fraction (LVEF) was observed in all subgroups. Mean LVEF of 60.7 ± 1.7% vs. 48.9 ± 6.2% at follow-up vs. preprocedural, respectively, in the younger patient group (**** *p* < 0.0001) and mean LVEF of 57.9 ± 7.2% vs. 42.5 ± 8.2% at follow-up vs. preprocedural, respectively, in the older patient group (**** *p* < 0.0001) (**A**). Mean LVEF of 60.2 ± 0.4% vs. 50.3 ± 4.8% at follow-up vs. preprocedural, respectively, in patients with mechanical prosthesis (** *p* = 0.0038) and mean LVEF of 58.7 ± 6.7% vs. 43.4 ± 8.2% at follow-up vs. preprocedural, respectively, in patients with biological prosthesis (**** *p* < 0.000001) (**B**). Mean LVEF of 58.9 ± 6.3% vs. 44.6 ± 8.4% at follow-up vs. preprocedural, respectively, in male patients (**** *p* < 0.00001) and mean LVEF of 60 ± 0% vs. 47 ± 6.7% at follow-up vs. preprocedural, respectively, in female patients (** *p* = 0.0065) (**C**). Results of two-way ANOVA are shown as mean ± standard deviation (SD).

Comparisons of LV remodeling according to type of implanted prosthesis, gender, and age are illustrated in Figure 2A–C. Considering that only a single case of PPM was detectable, analysis in this regard was not feasible. LVEF improvement was significant in all subgroups. Results of the two-way ANOVA showed that the only significant factor affecting LVEF increase was age (*p* = 0.0239). Younger patients tended to have less significant LVEF increase after treatment compared with patients over 60 years. The effect of the prosthesis type with a *p*-value of 0.069 shows a tendency towards more pronounced LVEF improvement in patients with biological valve replacement. Gender had no tendency to influence changes in LV function (*p* = 0.4908). No analysis on the effect of implanted valve size on reverse remodeling of the LV was performed since one of the only two patients receiving a 21 mm prosthesis suffered a periprocedural death.

## 4. Discussion

Reverse remodeling of the left ventricle following AVR has been demonstrated repeatedly [2,7,19]. In this context, aortic stenosis and subsequent hypertrophic remodeling, as well as the regression of LV hypertrophy and LV mass following surgery, have been described in detail [20]. Pressure overload enhances not only cardiomyocyte (CM) hypertrophy but facilitates perturbances in the extracellular matrix (ECM) too. The initial hypertrophy enables the preservation of cardiac output as an adaptive response. In the ECM, increased deposition of fibrillar collagen leads to increased myocardial stiffness, which, together with impaired CM relaxation, results in diastolic dysfunction. Adverse remodeling in response to volume overload, as in the case of AR, evokes distinct mechanisms. ECM degradation, loss of interstitial collagen, and CM apoptosis lead to dilatative LV remodeling with predominantly systolic dysfunction [21].

It is well described that prosthetic valve replacement generates completely different initial responses in these two pathologies. The abrupt cessation of mechanical obstruction in the case of a previously stenotic aortic valve elicits immediate regeneration of myocardial function and an uncomplicated weaning from cardiopulmonary bypass. The beneficial changes continue to be preserved over very long follow-up periods; in fact, the life expectancy of operated patients approximates that of the normal population [22,23,24]. On the other hand, the replacement of a regurgitant valve presents the already impaired and dilated LV with a suddenly difficult task. Not all ventricles are able to recover following surgical treatment; however, prognostic factors for early and long-term LV function improvement have only been partly identified [2].

Consistent with previous studies, our findings demonstrate significantly improved cardiac function and a reduction in left ventricular diameter following surgical treatment of aortic regurgitation (AR) in patients with impaired left ventricles. [7,25,26].

A novel and interesting observation is that biological prostheses initiated a more pronounced improvement in LV function than mechanical valves. Whereas research conducted in the 1990s indicated a more extensive LV reverse remodeling with stentless biological valves and aortic homografts than with stented biological or mechanical prostheses, little attention has been paid to comparing mechanical and (stented) biological prostheses, and even less research has been devoted to the particular patient cohort of AR with impaired LV function [27]. It is well known that stented biological and mechanical prostheses are associated with higher transvalvular gradients postoperatively than stentless valves and aortic homografts [28,29,30]. Their inferior hemodynamic features explain the less pronounced regression of LV impairment. However, a comparison of the much more commonly implanted mechanical and stented biological prostheses in terms of LV reverse remodeling is lacking. As younger patients tend to more frequently receive mechanical prostheses, the parallel findings in LVEF improvement are not surprising. Similarly to the younger patient group, we found less regression of LV impairment in patients receiving mechanical prostheses. However, another possible explanation would be in the mechanics of the different prostheses. Biological valves are tricuspid and mimic the native aortic valve in almost every anatomical and functional aspect. On the other hand, mechanical valves, even the new bileaflet designs with superior hemodynamics compared to the first caged-ball models, are still associated with complications resulting from non-physiological flow patterns. As the blood exits the LV through the three orifices of the mechanical valve, the cells travel with a higher velocity at the two lateral orifices than through the central one. The consecutive turbulent flow in the aortic root causes considerable shear stress. Moreover, as the leaflets close during diastole, some degree of leakage flow can be detected in different areas, but mostly in the hinge region of the prosthetic valve. The detrimental effects on red blood cells and thrombus formation are well described [31]. Even if the leakage volume seems to be unimportant, it may contribute to the less pronounced reverse remodeling. The greater extent of left ventricular reverse remodeling observed in our study compared to several other reports may be attributed to the proportionally higher use of biological prostheses.

As minimally invasive and interventional valvular replacement options are evolving, different treatment strategies become available for those young patients who are not willing to suffer the burden of lifelong blood thinning, its complications, and the audible noise associated with mechanical valves. The possibility of TAVI first, then upon the deterioration of the prosthesis, open surgical AVR, and years later another TAVI and eventually redo surgery, has been recently discussed as a feasible option for young patients refusing or not eligible for valve-sparing procedures or mechanical valves. The expense of all these prostheses should be weighed against the financial burden of complications of anticoagulative therapy or that of repeated interventions needed for patients following a Ross procedure [32].

## 5. Conclusions

Left ventricular reverse remodeling contributes to higher life quality as indicated by the low NYHA class and rare hospital readmissions. However, more data are needed on the degree of LV functional and geometrical improvement comparing the different types of valve prostheses to provide optimal treatment strategies, especially in young asymptomatic patients with aortic regurgitation.

## 6. Limitations

The primary limitation of this study is the relatively small sample size of patients included in the analysis. This constraint largely stems from the retrospective nature of data acquisition, which necessitated the exclusion of patients with insufficient preprocedural findings. Furthermore, the limited availability of comprehensive echocardiographic data further restricted our ability to perform a detailed analysis of left ventricular geometry or diastolic dysfunction. Consequently, these limitations may affect the generalizability of the study’s findings and highlight the need for more robust data in future research.

## Figures and Tables

**Table 1:** Demographics and preoperative data.

	n = 25
Age (years), mean ± SD (range)	59 ± 16.3 (29–79)
Male	20 (80%)
Diagnosis	
Chronic aortic regurgitation	23 (92%)
Acute aortic dissection	1 (4%)
Infective endocarditis	1 (4%)
Bicuspid aortic valve	8 (32%)
NYHA functional class	
I–II	11 (44%)
III–IV	14 (56%)
Comorbidities	
Hypertension	14 (56%)
Dyslipidemia	14 (56%)
COPD	6 (24%)
Diabetes mellitus	1 (4%)
Obesity (BMI > 30)	4 (16%)
Renal impairment ^1^	10 (40%)
Previous cardiac surgery	1 (4%)
EuroSCORE II mean ± SD (range), %	3.06 ± 5.00 (0.56–24.28)
LVEF ± SD (range), %	45.1 ± 8.01 (20–55)

SD: standard deviation, NYHA: New York Heart Association, COPD: chronic obstructive pulmonary disease, BMI: body mass index, LVEF: left ventricular ejection fraction, ^1^ Renal impairment: creatinine clearance < 85 mL/min (using the Cockroft–Gault formula) or on dialysis.

**Table 2:** Procedural aspects.

	n = 25
Concomitant procedures	17 (68%)
Isolated AVR	3 (12%)
Aortic root enlargement	3 (12%)
Root/ascending ± partial arch repair	2 (8%)
PFO closure	
Valve prosthesis model	
Mechanic	6 (24%)
SJM Regent	3 (12%)
ATS	2 (8%)
ON-X	1 (4%)
Stented bovine	18 (72%)
Edwards Perimount Magna	13 (52%)
SJM Trifecta	5 (20%)
Stented porcine Medtronic Hancock II	1 (4%)
Valve size	
21 mm	2 (8%)
23 mm	3 (12%)
25 mm	9 (36%)
27 mm	11 (44%)

AVR: aortic valve replacement, PFO: patent foramen ovale, SJM: St. Jude Medical, Inc. (Saint Paul, MN, USA), ATS: ATS Medical, Inc.

## Data Availability

Raw data are available upon request.
